# Assessing and coping with the financial burden of computed tomography utilization in Limbe, Cameroon: a sequential explanatory mixed-methods study

**DOI:** 10.1186/s12913-020-05830-1

**Published:** 2020-10-27

**Authors:** Joshua Tambe, Lawrence Mbuagbaw, Pierre Ongolo-Zogo, Georges Nguefack-Tsague, Andrew Edjua, Victor Mbome-Njie, Jacqueline Ze Minkande

**Affiliations:** 1 Post-Graduate School for Life Sciences, Health and Environment, The University of Yaoundé I, Yaoundé, Cameroon; 2 Division of Radiology, University of Buea, Buea, Cameroon; 3 Department of Health Research Methods, Evidence and Impact, McMaster University, Hamilton, Canada; 4 Biostatistics Unit, Father Sean O’Sullivan Research Centre, St Joseph’s Healthcare, Hamilton, Canada; 5 Department of Public Health, Biostatistics unit, Faculty of Medicine and Biomedical Sciences, The University of Yaoundé I, Yaoundé, Cameroon; 6 Higher Technical Teacher’s Training College Kumba, University of Buea, Buea, Cameroon; 7 Ministry of Public Health, Yaoundé, Cameroon

**Keywords:** Computed tomography utilization, Risk of financial hardship, Mixed-methods

## Abstract

**Background:**

There has been a significant increase in computed tomography (CT) utilization over the past two decades with the major challenges being a high exposure to ionizing radiation and rising cost. In this study we assess the risk of financial hardship after CT utilization and elaborate on how users adapt and cope in a sub-Saharan context with user fee for services and no national health insurance policy.

**Methods:**

We carried out a sequential explanatory mixed methods study with a quantitative hospital-based survey of CT users followed by in-depth interviews of some purposively selected participants who reported risk of financial hardship after CT utilization. Data was summarized using frequencies, percentages and 95% confidence intervals. Logistic regression was used in multivariable analysis to determine predictors of risk of financial hardship. Identified themes from in-depth interviews were categorized. Quantitative and qualitative findings were integrated.

**Results:**

A total of 372 participants were surveyed with a male to female sex ratio of 1:1.2. The mean age (standard deviation) was 52(17) years. CT scans of the head and facial bones accounted for 63% (95%CI: 59–68%) and the top three indications were suspected stroke (27% [95%CI: 22–32%]), trauma (14% [95%CI: 10–18%]) and persistent headaches (14% [95%CI: 10–18%]). Seventy-two percent (95%CI: 67–76%) of the respondents reported being at risk of financial hardship after CT utilization and predictors in the multivariable analysis were a low socioeconomic status (aOR: 0.19 [95%CI: 0.10–0.38]; *p* < 0.001), being unemployed or retired (aOR: 11.75 [95%CI: 2.59–53.18]; *p* = 0.001) and not having any form of health insurance (aOR: 3.59 [95%CI: 1.31–9.85]; *p* = 0.013). Coping strategies included getting financial support from family and friends, borrowing money and obtaining discounts from the hospital administration and staff.

**Conclusion:**

No health insurance ownership, being unemployed or retired and a low socioeconomic status are associated with financial hardship after CT utilization. Diverse coping strategies are utilized to lessen the financial burden, some with negative consequences. Minimizing out-of-pocket payments and/or the direct cost of CT can reduce this financial burden and improve CT access.

## Background

Medical imaging has undergone major technological advances in the last two decades with a significant rise in utilization and improved health outcomes for patients [1–9]. Computed tomography (CT) has particularly recorded an exponential increase in utilization in hospital emergency departments (EDs) in recent years [4, 10, 11]. Expanded clinical indications, self-referrals, defensive medicine, financial incentives for referring physicians and imaging specialists besides other factors account for the increase in CT utilization [4, 8, 12].

The utilization of multislice CT scanners in hospital EDs has been reported to improve on the management of injury, cancer, stroke and cardiac conditions [13–15]. Despite being the imaging “workhorse” in many EDs, CT utilization is not without challenges. CT imaging is a source of high exposure to ionizing radiation and there are conflicting reports on the association between radiation exposure to CT and the risk of malignancy [11, 16–21]. Also, the risk of adverse events from contrast material administration during CT procedures can further complicate its use [8, 22, 23]. Furthermore, the rising cost of advanced CT procedures raises both the direct and indirect cost of care [9–11, 24–26].

The cost of CT is a perceived barrier to utilization. According to the World Health Organization, high cost of healthcare services results in a drop in utilization [27]. In low-income settings with limited public financing for healthcare, the introduction of user fees can lead to decreased utilization with the poor having less access to healthcare services [28]. Healthcare services are mostly accessed through out-of-pocket (OOP) payments in resource-poor settings [27, 28]. OOP payments for services lead to unequal access to care [27, 29–33]. The economic consequences of OOP payments for healthcare services include spending high proportions of household income (catastrophic spending), borrowing money and “distress financing” such as the selling of assets, all of which can deepen poverty [27, 28, 34].

Socioeconomic status (SES) is an important determinant in the utilization of healthcare services with better access and improved health outcomes reported among people of high SES [35–38]. Conversely there is increased disease prevalence and mortality associated to a low SES [38–44]. The literature is however conflicting regarding the role of SES in CT utilization. Some authors have reported increased CT utilization among patients of high SES [3, 45] while others reported a high utilization rate in patients of low SES [6, 46]. There was no reported difference in CT utilization among SES categories in other studies especially when the clinical indication for CT was “clear-cut” [2, 45, 47]. These studies were carried out in high income countries with available public health insurance schemes.

Empirical studies on CT utilization have mainly focused on trends, determinants, cost of care and radiation exposure [1–3, 5–7, 10, 15, 19, 20, 45–61]. To the authors’ knowledge no study has specifically focused on the financial burden of CT utilization. Also, data on CT utilization in sub-Saharan Africa is scarce; one retrieved study reported on the appropriateness of CT utilization [62]. Through this study we sought to provide information on the financial burden of having to utilize CT in a sub-Saharan context with user fee for services and the absence of a national health insurance policy.

### Context

Public health facilities in Cameroon are stratified into tertiary, intermediate and peripheral levels based on the degree of sophistication of available technology and health services offered. The tertiary facilities (teaching and national referral hospitals) which are the most equipped in terms of equipment and medical specialists are found in the two chief towns of Yaoundé and Douala. Intermediate-level health facilities are termed Regional and District Hospitals and they serve as reference hospitals within administrative regions. In a bid to improve access to healthcare the government of Cameroon is face-lifting intermediate-level health facilities by creating specialized units such as hemodialysis and medical imaging centers that were previously only available in tertiary health facilities. Many medical specialists are being deployed to these hospitals which have mostly had general practitioners, obstetricians and general surgeons over the years.

The direct cost of CT scans in government-owned health facilities ranges from 42 to 175 US Dollars, slightly lower compared to private health facilities due to government subvention. According to a national demographic and health survey around 40% of the population live below the poverty line and 96–98% of the general population do not have any form of health insurance [63]. At the time of writing there is no national health insurance scheme in Cameroon. However some employers provide insurance policies for employees and there are private companies that encourage subscription as an individual or as a family. For the former type of insurance a certain amount of money is irreversibly deducted from the salary of the employee while for the latter the subscriber contributes an agreed sum of money regularly that is non-refundable. Cost coverage for healthcare services varies according to insurance policy ranging from 70 to 100% of the direct cost. There are also restrictions to healthcare services covered and some insurance policies impose a maximum amount of the direct cost they are willing to cover.

Given the context, it can be expected that CT utilization will be a challenge to people of low SES who may experience a heavier financial burden. Despite the expected challenges, CT scans are still being utilized as the need is often present at some point of care. To understand how CT users find a balance between their financial capability and the cost of CT scans in the study setting, we designed this mixed-methods study with main objective to assess the financial burden of CT utilization using risk of financial hardship as a proxy and to elaborate on how users adapt and cope. We formulated the following research questions: What factors are associated with the risk of financial hardship after CT utilization? How do some user characteristics translate into risk of financial hardship after CT utilization? How do CT users adapt and cope with having to use CT? How well does the qualitative phase augment the findings from the quantitative phase?

## Methods

### Study design

We used a sequential explanatory mixed-methods design [64]. Basically this paradigm generates information first through a quantitative phase which will have to be explained in greater detail with a qualitative phase. It permits the explanation of unexpected findings and generates multiple perspectives giving a more complete understanding of the phenomenon being studied [65–67]. The sequential explanatory design allows researchers to study variables in breadth and depth and with regard to the research questions for this study, it enables the quantitative assessment of the risk of financial hardship after CT utilization and further probing through in-depth interviews to understand how users adapt and cope [64]. We also expect the findings of this study to have implications for policy and practice in the study setting thus providing more grounds for adopting this paradigm which is compatible with the philosophical stance of pragmatism and critical realism [64, 68, 69].

**Fig. 1:**
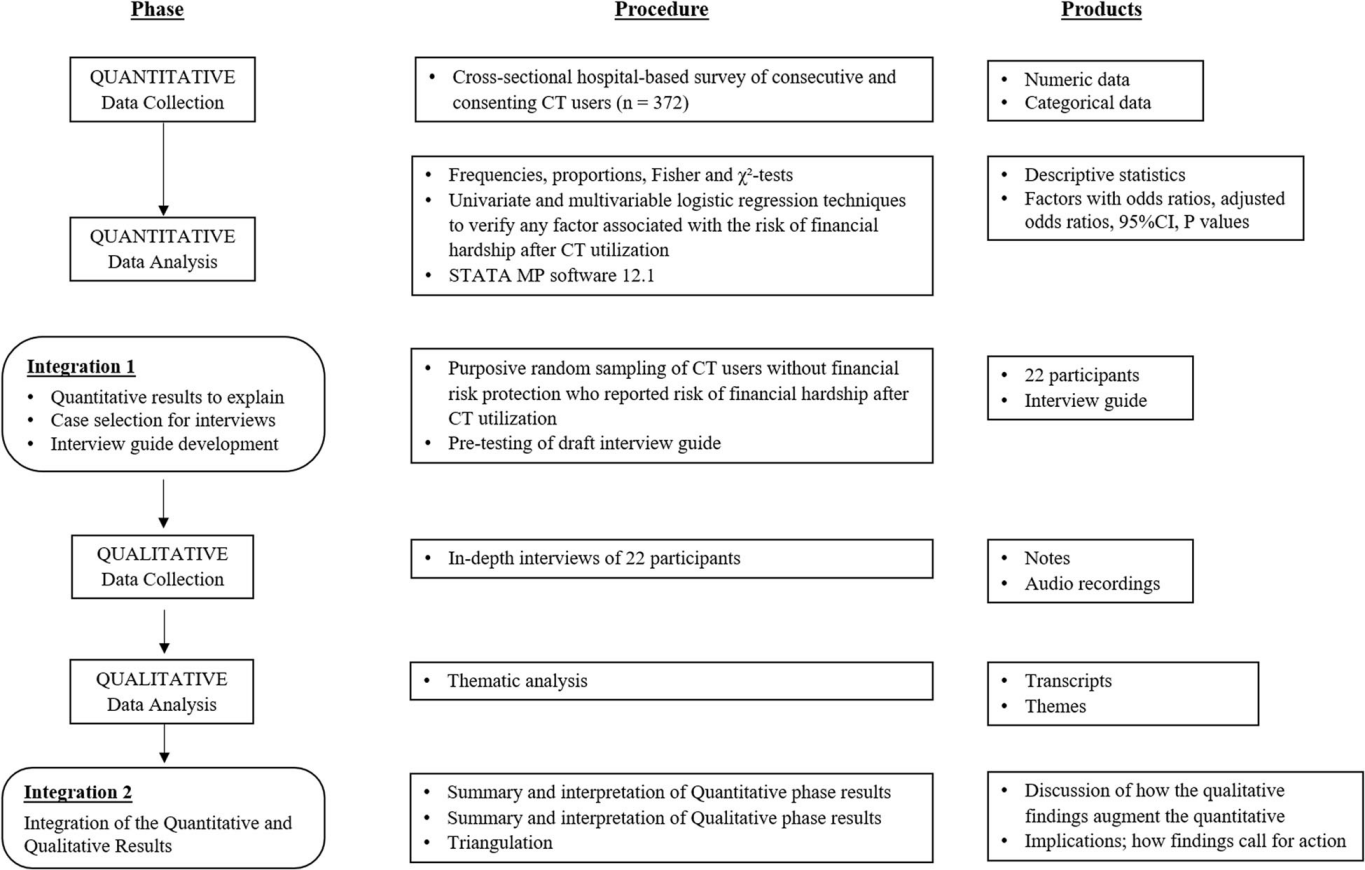
Study diagram

Theoretically the sequential explanatory mixed-methods paradigm gives more priority to the quantitative phase. The first author who was the principal investigator has a quantitative background and this paradigm was thus suitable. A summary diagram of the study is presented in Fig. 1.

### Ethics

Administrative authorization for the study was given by the South West Regional Delegation for Public Health (R11/MINSANTE/SWR/RDPH/82/786) and ethical approval was obtained from the Institutional Ethics Committee of Regional Hospital Limbe (002–03/2018/IEC-RHL) and The University of Yaoundé 1 (108/UY1/FMSB/VDRC/CSD).

### Study setting

This study was carried out at Regional Hospital Limbe in the South-West Region of Cameroon with the capacity of 200 beds. This facility was selected because it is the main intermediate-level referral health facility for the region and the only one with a functional CT scanner at the time the study was conducted. The first author also works in this facility as a radiologist making it convenient for data collection. Besides the 16-slice CT scanner there are other equipment such as digital radiography, mammography and a modern ultrasound scanner. The hospital has a 24-h emergency department, operation theatre, neonatology and pediatric units, antenatal clinic and maternity, medical and surgical wards for admission, and medical specialists across the main specialties.

### Participants

#### Quantitative phase

Consenting patients 18 years old and above who utilized CT at the Medical Imaging Center of Regional Hospital Limbe were consecutively enrolled from March 2018 to February 2019. Written informed consent was obtained from either the patient or the caregiver.

#### Qualitative phase

Participants were selected using a purposive random technique [70, 71]. These were patients who participated in the quantitative phase and reported to be at risk of financial hardship after CT utilization. Participant selection was done after quantitative data collection and analysis by carefully going through each quantitative data item. Eligible participants for the qualitative phase were phoned by the principal investigator and asked if they would like to participate in a phone interview. Appointments for the interviews were then taken for consenting participants. The participants for this phase were therefore a subset of participants from the quantitative phase [64]. It is worthy of note that the caregivers of some CT users were interviewed because the clinical state of these users did not permit them to be able to commit to an interview.

### Sample size

#### Quantitative phase

The minimum sample size was estimated using Cochran’s method for surveys [72]. The risk of financial hardship after CT utilization was the primary outcome and expressed as a binary categorical variable. With an alpha level of 0.05, a 5% error margin and a population variance estimate of 0.25, the estimated sample size was 385 participants. For a population of 1614 CT users (number of CT users during the first year of functioning), the sample size of 385 exceeds 5% of the population. Applying Cochran’s correction formula [72], the minimum return sample size was 310 participants. We further hypothesized a non-response rate of 20% and estimated inviting a total of 388 potential participants.

#### Qualitative phase

We invited 28 potential participants for in-depth interviews and 22 were actually interviewed. Two participants could not be reached by phone after several attempts, three others were not disposed for a conversation at the appointed time (one in a public transport vehicle and two in meetings), and one had just died and so the caregiver could not commit to the interview. This sample size was deemed satisfactory to explore participants’ experiences on CT utilization based on recommendations from Guest and Morse [73, 74]. According to these recommendations, 6 participants would be sufficient to explore experiences and 12 to obtain thematic saturation.

### Data collection

#### Quantitative phase

Structured interviews with standardized forms were used to collect data. The construction of the questionnaire was based on the study objectives. Content validation was used to ascertain the usefulness of the included items. After initial drafting by the first author, three other authors (LM, GN and POZ) took turns to cross-check the items rephrasing some, discarding others and suggesting some be modified and others introduced. Revised drafts of the questionnaire were pre-tested on some CT users for a few weeks to assess validity and clear any ambiguity. The final version of the questionnaire was consensually agreed upon by the authors and drafted in English and French. The outline was structured as follows: demographic attributes (age and sex), socialization patterns (region of origin, educational achievement; marital, employment and socioeconomic status; health insurance subscription), the presence of chronic illnesses, anatomic region scanned and clinical indication, payment method for CT and the risk of financial hardship after CT utilization. Socioeconomic status was assessed using household amenities score with each participant assigned to the quintile corresponding to their score. This tool has already been used in a previous study carried out in a similar setting [75]. The risk of financial hardship was determined using a self-reported question: “Do you have enough money to meet your needs including food, clothing, and payment of bills after paying for CT?” The options were “more than enough”, “just enough” and “less than enough” [76].

A trained research assistant with a BSc degree in sociology and fluent in English, French and the local pidgin-English language collected all the data. The standardized forms were available in English and French. The items were interpreted into the local pidgin-English language for participants who could best express themselves using that language. The data collector wore an identification badge and consenting participants were interviewed after CT had been done in an office made available for this purpose within the health facility. The principal investigator (PI) cross-checked all the forms after interviews for consistency.

#### Qualitative phase

A pre-established interview guide was used to collect qualitative data (interview guide included as an Additional file 1). This guide summarized important quantitative results to explain and study objectives that could only be explored using this method. Five typical respondents who were not part of the final sample were invited and interviewed by the PI (JT) using a first draft interview guide. Modifications were made to this draft after each interview and the revision following the last interview was adopted.

The PI (JT) called up potential participants on phone to schedule the in-depth interviews. Information shared during this first phone call included an overview of the talking points, the expected duration of the interview and subsequent appointment for a telephone interview at the convenience of the participant, should they consent to participate. All interviews were conducted by the PI in the preferred language of the participant. Whilst being open to a wide range of ideas during the interviews, the discussion was nevertheless kept in line with the study objectives. Permission to audio-tape the conversation was requested from the participants at the beginning of the interview.

### Data analysis

#### Quantitative phase

The information on the data forms were transcribed onto a Microsoft Excel® spreadsheet and analyzed using STATA® 12 (StataCorp, Texas, USA). Continuous variables were summarized using the mean and standard deviation. Categorical variables were summarized using frequencies, percentages and 95% confidence intervals (CI). The risk of financial hardship after CT utilization was categorized as a binary variable with the options “just enough” and “less than enough” merged into one category representing “risk” of financial hardship, while the option “more than enough” represented “no risk” of financial hardship. Merging of the categories was done without descriptive analysis or any particular consideration taken into account.

Sociodemographic variables were compared between respondents who reported risk of financial hardship and those who did not using Fisher’s exact and *chi*-squared tests where appropriate. Univariate and multivariable logistic regression techniques were used to determine if any factors were associated with the risk of financial hardship after CT utilization. In the multivariable modelling the following covariates were entered as a block: age, sex, marital status, educational achievement, employment status, SES, health insurance ownership and the presence of chronic illnesses (binary variable). Statistical tests were two-tailed and *p*-values less than 0.05 were considered statistically significant. Model fit was assessed using R^2^ statistic.

#### Qualitative phase

Thematic analysis was used to analyze the qualitative data given that the authors are more familiar with this method which is a “foundational” qualitative analytic method and also because of its flexibility as it can be independent of theory and epistemology [77, 78]. The audio recordings were reviewed many times by the PI so as to be familiar with the data. Word repetitions and indigenous categories were used to develop an initial categorization of recurring ideas [79]. The audio data were transcribed into written texts in English by the PI.

A theoretically-driven thematic analysis was adopted and guided by a six-phase tool provided by Braun and Clarke [77]. Initial manual coding of the texts was done by the PI. Identified themes were compared with initial categories. After multiple reviews of the coded texts identified themes were revised, redefined and reorganized. To improve on credibility and trustworthiness two other investigators (GN and AE) independently reviewed the transcripts and audio-recordings for accuracy. The final list of categorized themes was consensually agreed by the authors.

### Integration of findings

The findings from both strands of the study were integrated to gain a more complete understanding that is “greater than the sum of the parts” [65]. Triangulation was used to integrate the findings [80]. Each finding of interest from either the quantitative or qualitative phase of the study was compared and contrasted with the findings of the other phase for convergence, complementarity, dissonance and “silence” [80]. “Silence” implied the particular finding could only be explored during a particular phase of the study.

## Results

### Participant characteristics

A total of 372 participants were surveyed of which 167 (45%) were females. The mean age (standard deviation) was 52 (17) years with age range 18 to 92 years. Table 1 summarizes participant characteristics.

**Table 1:** Characteristics of study participants with comparison between those who did/didn’t report risk of financial hardship

Variables	Frequency (%)	*P* value*
Age group (years; *N* = 344)		0.088
18–24	21 (5.7)	
25–34	51 (13.7)	
35–44	55 (14.8)	
45–54	76 (20.4)	
55–64	55 (14.8)	
> 65	114 (30.6)	
Sex (*N* = 372)		0.069
Female	167 (44.9)	
Male	205 (55.1)	
Marital status (*N* = 372)		0.277
Married	232 (62.7)	
Single	83 (22.3)	
Widow(er)	42 (11.3)	
Divorced	11 (3.0)	
Living in union	4 (1.1)	
Educational achievement (*N* = 347)		< 0.001
< O level	158 (45.5)	
O level or equivalent	61 (17.6)	
A level or equivalent	64 (18.4)	
Degree or equivalent	45 (13.0)	
Master & above	19 (5.5)	
Employment status (*N* = 294)		0.001
Employed, with contract	82 (27.9)	
Employed, with no contract	2 (0.7)	
Self-employed	115 (39.1)	
Unemployed	12 (4.1)	
Retired	83 (28.2)	
Socioeconomic status quintiles (*N* = 370) from the lowest (1) to the highest (5)		< 0.001
SES quintile 1	74 (20.0)	
SES quintile 2	75 (20.3)	
SES quintile 3	75 (20.3)	
SES quintile 4	79 (21.4)	
SES quintile 5	67 (18.1)	
Health insurance ownership (*N* = 370)		< 0.001
Yes	38 (10.3)	
No	332 (89.7)	
Number of deponents (*N* = 272)		0.425
0 to 3	117 (43.0)	
4 to 6	97 (35.7)	
7 and above	58 (21.4)	
Presence of chronic illnesses (*N* = 369)		0.232
Yes^a^	130 (35.2)	
No	239 (64.8)	

*P values obtained using Fisher’s exact and χ^2^ tests

^a^ Included in this category are hypertension, diabetes, peptic ulcer disease, cancer, chronic kidney disease, hyperprolactinemia

### Anatomic region scanned and clinical indications

CT scans of the head and facial bones accounted for 236 out of 372 scans (63% [95%CI: 59–68%]) and the top three indications were suspected stroke, transient ischemic attack or hypertensive emergency (27% [95%CI: 22–32%]), trauma (14% [95%CI: 10–18%]) and persistent headaches, blurred vision or suspected space-occupying lesion (14% [95%CI: 10–18%]). Tables 2 and 3 summarize the anatomic regions scanned and the clinical indications respectively.

**Table 2:** Anatomic regions scanned

Anatomic region scanned (*N* = 372)	Frequency (%; 95%CI)
Head + facial bones	236 (63.4; 58.5–68.3)
Abdomen	46 (12.4; 9.0–15.7)
Spine	41 (11.0; 7.8–14.2)
Chest + abdomen	14 (3.8; 1.8–5.7)
Chest	10(2.69; 1.0–4.3)
Angiograms	7 (1.88; 0.5–3.3)
Neck region	4 (1.08; 0.0–2.1)
Multiple regions	14 (3.77; 1.8–5.7)

**Table 3:** Indications for scanning per anatomic region

Indications for CT scan^a^	Frequency (%; 95% CI)
Head & facial bones	
Suspected stroke/transient ischemic attack/hypertensive emergency	86 (27.0; 22.2–31.9)
Trauma	45 (14.1; 10.3–18.0)
Persistent headaches, blurred vision, suspected space-occupying lesion	44 (13.8; 10.0–17.6)
Chest	
Suspected pulmonary embolism	7 (2.2; 0.6–3.8)
Chronic cough	4 (1.2; 0.0–2.5)
Tumor workup	2 (0.6; 0.0–1.5)
Abdomen/Pelvis	
Pain, acute abdomen	18 (5.7; 3.1–8.2)
Suspected tumor, mass	22 (6.9; 4.1–9.7)
Urinary symptoms	15 (4.7; 2.4–7.0)
Spine	
Back ache (severe, chronic, persistent)	28 (8.8; 5.7–11.9)
Suspected cord compression	9 (2.8; 1.0–4.6)
Trauma	6 (1.9; 0.4–3.4)

^a^Data available for 318 respondents

### Risk of financial hardship after CT utilization

Among study participants, 246 out of 344 (72% [95%CI: 67–76%]) declared having “just enough” or “less than enough” money to cater for their bills, food and clothing after paying for the scan, indicating risk of financial hardship. A hundred and two respondents out of 370 (28% [95%CI: 23–32%]) reported to have negotiated for CT direct cost reduction with 44 (43% [95%CI: 34–53%]) doing so formally through the hospital Social Services or the administration and 58 (57% [95%CI: 47–66%]) illegally through hospital staff directly related with the provision of care. Table 4 shows the relationship between some selected variables and risk of financial hardship in univariate and multivariable analyses.

**Table 4:** Risk of financial hardship after CT utilization

Variables	Univariate		Multivariate	
	Odds ratio (95% CI)	*P* value	Adjusted Odds ratio (95% CI)	*P* value
Age group (years; *N* = 344)				
18–24	1.39 (0.42–4.64)	0.589	0.63 (0.10–3.97)	0.624
25–34	ref		ref	
35–44	2.47 (1.00–6.08)	0.049	4.22 (1.18–15.11)	0.027
45–54	2.26 (1.02–5.01)	0.046	3.10 (0.80–10.66)	0.073
55–64	2.47 (1.00–6.08)	0.049	0.41 (0.09–1.78)	0.234
> 65	1.17 (0.58–2.34)	0.665	0.07 (0.01–0.48)	0.007
Sex (*N* = 344)				
Female	1.55 (0.96–2.50)	0.070	1.25 (0.60–2.60)	0.553
Male	ref		ref	
Marital status (*N* = 344)				
Married/living in union	ref		ref	
Single/divorced/widow	1.36 (0.83–2.24)	0.222	2.05 (0.80–5.23)	0.135
Educational level (*N* = 319)				
≤ O level	2.42 (1.44–4.06)	0.001	2.07 (0.90–4.76)	0.087
> O level or equivalent	ref		ref	
Employment status (*N* = 266)				
Employed (formally, informally, self)	ref		ref	
Unemployed/Retired	1.05 (0.60–1.86)	0.855	11.75 (2.59–53.18)	0.001
SES (*N* = 342)	0.20 (0.12–0.34)	< 0.001	0.15 (0.07–0.33)	< 0.001
Health insurance ownership (*N* = 342)				
Yes	ref		ref	
No	6.28 (2.73–14.45)	< 0.001	5.56 (1.74–17.76)	0.004
Chronic illnesses (*N* = 369)				
Yes	1.36 (0.82–2.24)	0.233	2.07 (0.93–4.58)	0.074
No	ref		ref	

Model *R*^*2*^ = 0.2685; *p* < 0.001; *ref*, reference category

### Qualitative findings

Quantitative phase participants who reported to be at risk of financial hardship were purposively selected for in-depth interviews. None of the selected participants reported any health insurance subscription. The interviews lasted between 10 and 18 min. Table 5 summarizes some characteristics of the interviewees.

**Table 5:** Characteristics of interviewees

Number of participants	22
Male	9
Female	13
Mean age (SD), years	49.7 (9.9)
Employment status of the patients	
Self-employed	8
Retired	8
Unemployed	3
Employed with a formal contract	3
Interviewee	
Patient	16
Caregiver	6

Three main themes related to CT utilization were identified: I) coping with CT utilization, II) unavoidability of CT utilization and III) deterrents to CT utilization and missed opportunities. There were three and two subthemes for themes I and III respectively.

### Coping with CT utilization

#### Family support

Some participants reported that close family relatives were called to financially assist them. Those called up were not limited to the nuclear family and even included close friends. In the study context regular use of the word “family” goes beyond blood ties. The excerpts below from three participants illustrate:

“… my husband is a logger and works for himself … since he has been down with sickness it is not easy so I have to support him financially … I sell pea nuts” (P01; caregiver of patient in late 30’s)

“We had to pay for the scan. She is not working and the doctor had planned to operate her … where was she supposed to get the money from?” (P08; caregiver of female patient).

“… we came prepared … my mother paid for everything” (P09; caregiver of patient in mid-50’s)

Despite many participants’ acknowledgement of receiving help from family, some nevertheless stated they had to “dig deep” into their savings to pay for the cost of CT all by themselves. These participants also claimed in doing so they felt a “vacuum” in their reserves but considered it necessary so that they could be diagnosed and properly treated.

#### Exonerations

Some participants declared they benefitted from some sort of discount. This happened through mainly two pathways: fee reduction approved by hospital administration or Social Services, and through staff directly involved with the provision of services. The former was reported by participants who either claimed to personally know some members of the hospital administration, belonged to the same ethnic group, attended same church, or upon recommendation from a political or local administrative authority. The benefits ranged from paying nothing at all to a 75% discount on the direct cost. Concerning the second pathway of fee reduction, participants admitted they paid money directly to hospital staff for services and often at a discount after some negotiations. This practice is illegal but participants believed was a win-win situation; they benefitted from direct cost reduction and some fast-tracking to obtain their results while the healthcare staff had a supplementary income. The accounts below from four participants illustrate:

“I had to give part of the money for the scan to the “nurse” first … I told him I cannot run away since my patient is admitted in the hospital” (P18; caregiver of patient in early 50’s)

“… pension is how much? The government doesn’t know what the people are going through … as a senior citizen I had to ask the director for a reduction and he cut the cost by two” (P02; retired, female)

“… I know the director personally … so I went to him [director] for consultation and he prescribed the scan himself and asked me to pay 50% of the cost” (P10; male in early 30’s)

“I explained my situation [financial] to the person I met who offered to help me … so I gave him what I had” (P11; female in mid-40’s)

#### Borrowing money

Having to borrow money from neighbors, friends and small common interest groups was also reported as a means of raising money to pay for CT when the need arose.

“I was pushed to borrow money because I was not feeling fine at all” (P04; unmarried female participant)

“I had to stretch my hands to my neighbors … I am on a loan” (P21; male in mid-50’s)

### Unavoidability of CT utilization

Despite reporting to be at risk of financial hardship after CT utilization, CT is still being utilized. One of the reasons why CT was promptly done was because of reported pain. Also, some participants believed appropriate medical care would only be administered after CT scan was performed. Furthermore some CT users had the understanding that CT scanning was necessary to determine the cause of ill health and therefore guide treatment. To others still CT scan was considered to be a kind of “one-stop-shop” test for the entire body and was expected to “reveal any anomaly” besides the present complaint. The excerpts below from two participants illustrate:

“My patient was feeling some ‘hot pains’ so we had no choice but to run up and down to mobilize the funds to get the scan done” (P01; caregiver of patient in late 30’s).

“… I am feeling very bad … cannot walk right now … I had to do it [CT] so that my entire body could be properly checked” (P06; male in mid-60’s)

### Deterrents to CT use and missed opportunities

#### Fear

Some participants reported not showing up for CT scan despite having received a prescription from a healthcare provider (for clients who had to do a repeat CT) because of fear. The reasons were varied: no money as previous experience showed the cost was substantial, resentful attitude of hospital staff as clients feared being ridiculed should they show up with insufficient funds, the scare of the equipment as patients are left alone inside the room, and also the fact that the machine uses x-rays which should have a long term effect in “reducing the lifespan” according to some participants.

“… money issues otherwise we were supposed to have done another CT scan following treatment …” (P01; caregiver of patient in late 30’s)

“If you dare go to hospital without money do you know what the staff can do to you?” (P03; female in early 60’s)

“The machine is scary … didn’t like being left alone in the room … not my first time doing CT scan and I am already afraid of the effect of the rays on my body” (P17; male in mid-50’s)

#### Ignorance

Some participants were not aware that CT services could be provided in emergency situations before the financial obligations were met. They refused to believe when this fact was explained and relied on anecdotes and past experiences with using healthcare services where pre-payment was mandatory. Also there was no knowledge of the Social Action Service, a department within the hospital facility that identifies paupers within the local community to offer them some fee reduction so that they can use needed healthcare services.

## Discussion

In the quantitative analysis a low socioeconomic status, being out of work (unemployed or retired) and not having any form of financial protection for health were associated with risk of financial hardship after CT utilization. The qualitative data revealed different coping strategies to reduce the burden of OOP payments for CT. These coping mechanisms have consequences for the health facility such as the loss of income through wanton exonerations and illegal financial practices by some staff. In addition to coping with CT utilization, the qualitative phase further identified potential barriers to CT utilization.

If a lower socioeconomic status would mean less financial viability, then both the quantitative and qualitative strands of the study agree that persons with a low SES are at risk of financial hardship after CT utilization. This finding indicates an association between SES and CT utilization, similar to reports by some authors [3, 45]. The age groups 35–44 and 45–54 years were associated to an increased risk of financial hardship after CT utilization. This finding can be explained by a possible increase in family and social responsibilities at this period of life. However, being 65 years old and above was a protective factor to the risk of financial hardship after CT utilization. Brown and colleagues reported the contrary with an increased likelihood of economic insecurity among men getting towards retirement [81].

There was further agreement on the association between employment status and the risk of financial hardship after CT utilization by both the quantitative and the qualitative phases of the study. Being unemployed, retired or temporarily out of work was linked with the inability to pay for the direct cost of CT. This finding was consistent with reports of associations between high unemployment and temporary work with low CT utilization by De Basea and colleagues [47].

The qualitative phase of this study highlights the unavoidability of CT utilization at some point of healthcare. The role of CT in patient management cannot be undermined and it will therefore have to be utilized if the need is perceived, whatever the difficulties expressed by prospective users. Family support was found to facilitate CT utilization. Other cost-reduction methods such as discounts obtained from the hospital administration and healthcare staff also reduced the financial burden of CT utilization. However, the possibility of a negative exploitation of the discount scheme through abuse of influence and power coupled with illegal financial transactions between service users and hospital staff can only lead to further loss of public revenue.

To effectively utilize healthcare services prospective users have to be able to approach the facilities where services are provided and get all the required information. Inability to overcome previous bad experiences with health services and ignorance of present possibilities can result to the non-utilization of needed services.

In this study the qualitative phase complements and emphasizes the quantitative findings permitting a broader perspective that cuts across both strands of the study. This study gives insight into the financial barriers to CT utilization and potential impact in the study context, reveals threats to the current organizational culture such as the leakage of public revenue and suggests avenues to improve utilization.

Improving access to healthcare services is an important step towards achieving universal health coverage [82]. This can be facilitated by protecting prospective service users from the negative impact of unaffordable OOP payments for healthcare services. OOP payments for healthcare services cause financial hardship, deter people from seeking or continuing care, can push entire households further below the poverty line (impoverishment) or require they forego other basic necessities [27, 83].

### Strengths and limitations

To improve upon the validity and credibility of the findings, content validation of data collection tools and pre-testing were done. Data collection was done by a single individual for each phase of the study to achieve consistency. The principal investigator supervised the research assistant during data collection. Also, there was an independent review of data forms and transcripts by investigators with a different research background (social sciences and public health). Furthermore, triangulation of methods with the use of both quantitative and qualitative designs to study the same phenomenon consolidates the findings.

Some limitations to this study exist. Firstly, the main outcome of the quantitative phase was self-reported and so reporting bias may have occurred. There are also concerns of reflexivity and power as the qualitative in-depth interviews were conducted by the PI, whose worldview and intuition could have influenced the reporting. Nevertheless we believe the absence of physical contact during the in-depth interviews might have limited the power gradient to some extent. Also, some participants could not be reached by phone or were not disposed for an interview when calls were made. We further report that no software was used for qualitative data analysis given the diversity of languages used for the interviews and unavailability of appropriate software.

## Conclusion

As findings from this study no health insurance ownership was associated to the risk of financial hardship after CT utilization. People who were unemployed or retired and of low socioeconomic status were also found to be at risk of experiencing a heavier financial burden after CT utilization. Given that CT utilization can be unavoidable at some point of care, some coping strategies to curb the financial burden following its utilization are being practiced. We however note that some of these coping strategies have negative consequences for the health facility and the government as the main financial stakeholder for the public health sector.

It is our opinion that measures to minimize OOP payments for CT can reduce the financial burden associated to its utilization. Furthermore a reduction of the direct cost of CT (user fee) might also improve financial accessibility (affordability). If public hospital revenue must be secured then hospital administrations have to put in place a strategy to check on illegal financial practices. We therefore recommend that the government in its role to protect its citizens should consider all the available evidence and take necessary action to improve on financial accessibility to healthcare services such as CT.

## Supplementary Information

**Additional file 1.** In-depth interview guide.

## Data Availability

The dataset supporting the conclusions of the quantitative phase of this article is available at Mendeley Data, 10.17632/r4dmt58v3r.1. The qualitative data contains sensitive information and can be available from the corresponding author upon reasonable request.

## Abbreviations

CT: Computed tomography
OOP: Out-of-pocket
SES: Socioeconomic status
PI: Principal investigator
CI: Confidence interval
