# Supplementary material for: Assessing and coping with the financial burden of computed tomography utilization in Limbe, Cameroon: a sequential explanatory mixed-methods study
Source: BMC Health Serv Res. 2020 Oct 27;20:981. doi: 10.1186/s12913-020-05830-1 (PMC7590681; doi:10.1186/s12913-020-05830-1)
Supplement: Supplementary file 1 — Additional file 1. In-depth interview guide. [file 12913_2020_5830_MOESM1_ESM.docx]

**In-depth interview guide**

- How often within the past twelve months have you had health problems for which CT was requested?
- How do you typically raise money to cater for your health needs?
- Describe how it was like raising the money and paying for this CT scan.
- What particular difficulties did you encounter and how did you go about them?
- How do you think some aspects of your current situation (such as family life, job situation, educational achievement, lack of health insurance, current income) affected the way you had to go about getting the scan done?
- How much time did it take for you to get the scan done from the day it was prescribed, and why so?
- How do you feel about catering for your basic needs for the days ahead?
- What, in your opinion, are the opportunities which if in place would make it easier for you to obtain CT or feel less financial stress after?
